# Tree shrew as a new animal model for musculoskeletal disorders and aging

**DOI:** 10.1038/s41413-024-00367-z

**Published:** 2025-01-02

**Authors:** Xiaocui Wei, Honghao Li, Jingyang Qiu, Jianlin Jiao, Xiongtian Guo, Gaosheng Yin, Ping Yang, Yi Han, Qiongzhi Zhao, Hao Zeng, Zhi Rao, Xuefei Gao, Kai Li, Pinglin Lai, Sheng Zhang, Chengliang Yang, Di Lu, Xiaochun Bai

**Affiliations:** 1 State Key Laboratory of Organ Failure Research, Department of Cell Biology, School of Basic Medical Sciences, Southern Medical University, Guangzhou, 510515 China; 2 Department of Histology and Embryology, School of Basic Medical Sciences, Southern Medical University, Guangzhou, 510515 China; 3 Guangdong Provincial Key Laboratory of Bone and Joint Degeneration Diseases, The Third Affiliated Hospital of Southern Medical University, Guangzhou, 510630 China; 4 Yunnan Key Laboratory of Stem Cell and Regenerative Medicine, School of Rehabilitation, Kunming Medical University, Kunming, 650500 China; 5 Department of Physiology, School of Basic Medical Sciences, Southern Medical University, Guangzhou, 510515 China; 6 Guangxi Key Laboratory for Biomedical Material Research, Department of Orthopedics, Affiliated Hospital of Youjiang Medical University for Nationalities, Baise, 533000 China

**Keywords:** Bone, Bone quality and biomechanics

## Abstract

Intervertebral disc degeneration (IDD), osteoarthritis (OA), and osteoporosis (OP) are common musculoskeletal disorders (MSDs) with similar age-related risk factors, representing the leading causes of disability. However, successful therapeutic development and translation have been hampered by the lack of clinically-relevant animal models. In this study, we investigated the potential suitability of the tree shrew, a small mammal with a close genetic relationship to primates, as a new animal model for MSDs. Age-related spontaneous IDD in parallel with a gradual disappearance of notochordal cells were commonly observed in tree shrews upon skeletal maturity with no sex differences, while age-related osteoporotic changes including bone loss in the metaphyses were primarily presented in aged females, similar to observations in humans. Moreover, in the osteochondral defect model, tree shrew cartilage exhibited behavior similar to that of humans, characterized by a more restricted self-healing capacity compared to the rapid spontaneous healing of joint surfaces observed in rats. The induced OA model in tree shrews was highly efficient and reproducible, characterized by gradual deterioration of articular cartilage, recapitulating the human OA phenotype to some degree. Surgery-induced IDD models were successfully established in tree shrews, in which the lumbar spine instability model developed slow progressive disc degeneration with more similarity to the clinical state, whereas the needle puncture model led to the rapid development of IDD with more severe symptoms. Taken together, our findings pave the way for the development of the tree shrew as a new animal model for the study of MSDs and aging.

## Introduction

Musculoskeletal disorders (MSDs) are one of the most prevalent chronic conditions and represent a major international public health challenge. Intervertebral disc degeneration (IDD), osteoarthritis (OA), and osteoporosis (OP) are common MSDs with similar age-related risk factors.^1^ Low-back pain, mostly associated with IDD, is the leading cause of total global years lived with disability,^2,3^ while among the elderly population, OA is the most common cause of reduced mobility.^4^ With the mean population age rising, MSDs are expected to further increase medical resource investment and societal burden. Nevertheless, current treatments essentially constitute either palliative care, failing to address the underlying disease progression^5,6^ or else involve invasive procedures with adverse effects,^7,8^ leading to a substantial need for disease-modifying therapies.^9^

Research on animal models has enhanced our understanding of the complex pathophysiology of MSDs, thus providing potential translational therapy targets. However, the success of translating these observations and the demonstration of clear efficacy in clinical trials have been poor,^10,11^ partially due to the lack of clinically-relevant experimental models. Although non-human primates can accurately simulate the characteristics of the human musculoskeletal system, they also suffer from major limitations as experimental platforms due to high costs, operational difficulties, long experimental cycles, and ethical restrictions.^12,13^ Rodents are the most commonly-used animal models with many advantages such as short breeding cycles and low housing costs. However, the genetic differences between rodents and humans are substantial, making the clinical translation of many experimental achievements difficult.^14^ In addition, the growth plates do not close in rodents, which likely increases the intrinsic healing potential and interferes with the applied treatment.^15^ Large animal models such as the goat or the horse more closely resemble the human musculoskeletal system compared to smaller animal models,^16,17^ but it is usually not fiscally feasible or practical to conduct initial experiments in larger species. Overall, an ideal animal model that combines all the desirable properties has not yet been found. Considering the heterogeneous and multifactorial profile of MSDs, developing new animal models or combining several different animal models is necessary to provide greater insight into disease manifestation.

The tree shrew (*Tupaia belangeri*) is a small mammal with a variety of unique characteristics making it suitable for use as an experimental animal, including moderate body size, short life cycle (4–6 months from birth to adulthood), high fecundity (2–6 offspring born per cycle), low cost of maintenance, and most importantly, a claimed close affinity to primates.^18,19^ Whole-genome phylogenetic analysis has confirmed that the tree shrew is highly homologous to humans.^20^ Further, it has a much closer relationship to primates than rodents, guinea pigs or rabbits at the anatomical, behavioral, and evolutionary levels.^21–23^ Moreover, the success of CRISPR/Cas9-mediated gene editing in tree shrew spermatogonial stem cells has provided opportunities for mechanistic in vivo study,^24^ facilitating the wider application of this animal in biomedical research. Due to their genetic similarity to humans and the availability of tools for population genetics, numerous models based on tree shrews have been thoroughly explored in studying myopia,^25–27^ hepatitis virus infections,^28,29^ depression,^30^ aging,^31^ learning behaviors as well as social stress.^32^ Even though an osteoporotic model in tree shrews has previously been described,^33,34^ thorough systematic evaluation of its potential suitability and wider application in MSDs and aging are still scarce.

In the present study, the age-related IDD and osteoporotic changes observed in tree shrews were characterized. We further revealed that growth plate fusion occurred in tree shrews after young adulthood, which contributed to their much more limited intrinsic regenerative capacity than rats in models of osteochondral defects. The establishment of OA and IDD models further boosted the wider prospective application of the tree shrew, highlighting its potential for use as a viable animal model of MSDs.

## Results

### Tree shrews developed aged-related IDD with a gradual disappearance of notochordal cells

**Fig. 1:**
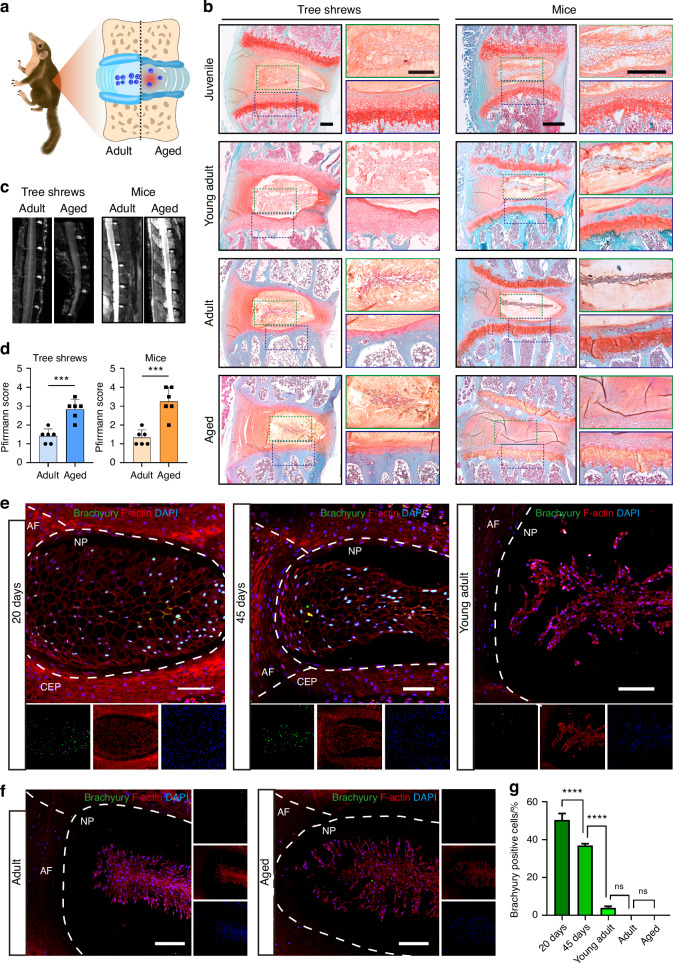
Lumbar intervertebral discs in tree shrews developed aged-related degeneration in parallel with a gradual disappearance of notochordal cells. **a** Schematic of the intervertebral disc in adult and aged tree shrews. **b** Representative Safranin O/Fast Green (Saf-O/FG) staining of disc tissue in tree shrews (left) and mice (right) at different ages (juvenile tree shrews, 45 days; young adult tree shrews, 4.5 months; adult tree shrews, 12 months; aged tree shrews, 3.5 years; juvenile mice, 21 days; young adult mice, 3 months; adult mice, 6 months; aged mice, 18 months). Scale bar, 250 μm. **c** Representative MRI images of the lumbar vertebrae of adult and aged tree shrews and mice. **d** Quantitative analysis of MRI, *n* = 6 tree shrews and *n* = 6 mice. **e** Representative immunofluorescence (IF) images of disc tissue in juvenile (left, 20 days; middle, 45 days) and young adult (right, 4.5 months) tree shrews. Scale bar, 100 μm. **f** Representative IF images of disc tissue in adult (12 months) and aged (3.5 years) tree shrews. Scale bar, 100 μm. **g** Quantification of IF stains (**e**, **f**) for brachyury, *n* = 3. Data are expressed as the mean ± SD, *P < 0.05; **P < 0.01; ***P < 0.001; ****P < 0.000 1; ns, *P* non-significant

The human intervertebral disc is known to degenerate with age, which is one of the main factors associated with IDD.^35^ To evaluate the potential suitability of the tree shrew model for mimicking human pathology, we systematically examined the changes of disc architecture and cell type in tree shrews at different ages. Consistent with a previous report,^36^ we observed that tree shrews had six lumbar vertebrae with similar vertebral length and increasing antero-posterior diameter of the endplates. Notably, the vertebral growth plates initiated closure in young adult tree shrews (4.5 months), unlike in mice where they remained open across all age groups examined (ages ranging from 21 days to 18 months) (Fig. 1a, b). Moreover, growth plates in tree shrews were restricted to the base of the cartilage endplate (CEP) corresponding to those observed in humans, in which the base of the CEP acts as the growth region for the vertebral body (Fig. 1b). In contrast, in mice and many other species (including sheep and cows), growth plates are found within the vertebral bodies.^37^ Further MRI and histological analysis of changes in the intervertebral disc in skeletally-mature tree shrews was performed. Compared to adult tree shrews (12 months), both male and female aged tree shrews (3.5 years) exhibited mild degenerative changes in their discs, including development of clefts/fissures in the annulus fibrosus (AF), gradual loosening of the nucleus pulposus (NP) cell cluster and reduction of cell number (Fig. 1a, b), a decrease in disc space height, and higher Pfirrmann score (Fig. 1c, d). Age-related IDD was also observed in 18-month-old mice, characterized by a loss of disc height and space, replacement of the NP matrix with fibrous cartilaginous tissue, as well as thickening and central ingrowth of AF fibers (Fig. 1b).

Human notochordal cells are lost with skeletal maturity, resulting in an imbalance in NP homeostasis and suspected to be a major event in early IDD.^35^ We next evaluated cell morphology and expression of the notochordal cell marker brachyury in NP cells of tree shrews. Of note, in juvenile tree shrews, the NP was highly cellular, containing large vacuolated notochordal cells with a dense actin cytoskeleton and high expression of brachyury (Fig. 1e). With aging, we found a progressive decline in vacuolated cells and a concomitant increase in smaller, chondrocyte-like cells. The expression of brachyury was barely detectable after young adulthood (Fig. 1e–g). These results indicated that tree shrews resembled humans in that the notochord cells present at birth rapidly decline in number early in life, which may confer advantages for the pathological study of intervertebral disc aging and disease progression.

In summary, lumbar intervertebral discs in tree shrews displayed degenerative characteristics with aging in parallel with a gradual disappearance of notochordal cells.

### Female tree shrews were more susceptible than males to osteoporotic changes at the geriatric stage

Bone loss is a universal human phenomenon and causes fragility fractures, with the risk increasing with age. Age-related OP in tree shrews has not been described. In this study, the long bones from tree shrews of different ages–juvenile (45 days), young adult (4.5 months), adult (12 months), and aged (3.5 years)–were isolated to assess their morphologic, microstructural, and mechanical features. Across the four examined age groups in male tree shrews, there was a generally increasing trend in femur length, diameter, and mechanical properties (tibia maximum load) with age (Fig. S1a, b, d). There were accompanying increases in bone mineral density (BMD), trabecular thickness (Tb.Th) and cortical bone thickness (Ct.Th) based on micro-architectural parameters analyzed by micro-computed tomography (micro-CT) (Fig. S1c, f, g). Histological analysis revealed that the bone was undergoing mineralization as illustrated by the extensive presence of cartilage throughout the distal head in the juvenile tree shrews (Fig. S1e). Growth plate fusion occurred in the young adult group. Adult tree shrews had fully mineralized bones and displayed hallmarks of a mature femur, with fully formed trabecular bone (Fig. S1e).

**Fig. 2:**
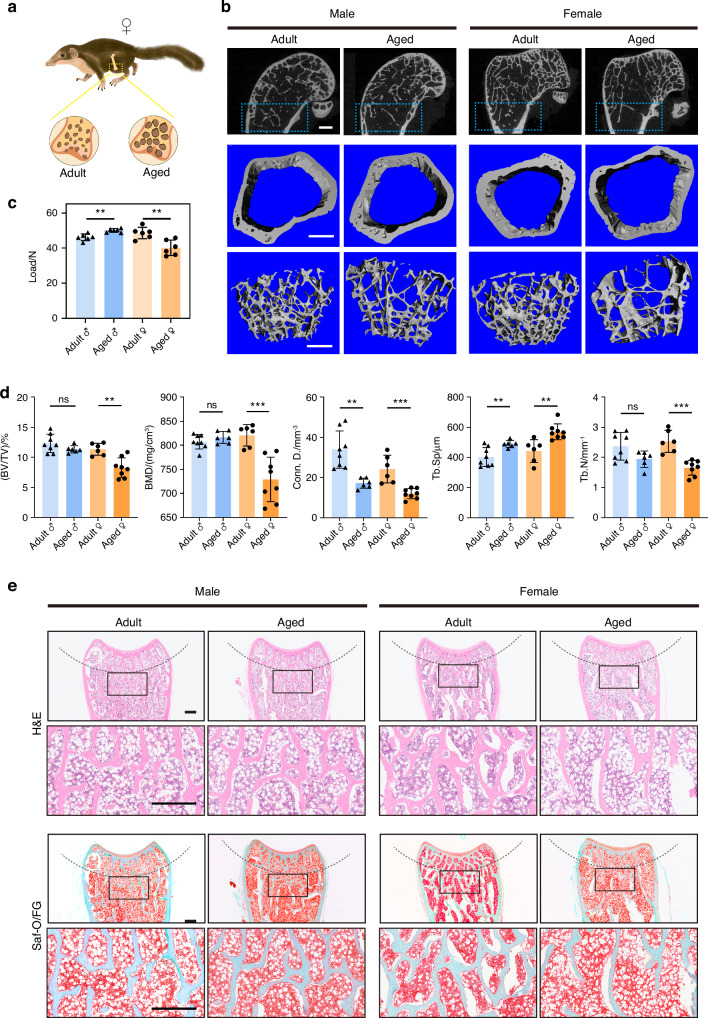
Aged female tree shrews displayed osteoporotic changes. **a** Schematic of the distal metaphysis in adult and aged tree shrews. **b** Representative 3D reconstructed micro-CT images showing the distal femoral metaphysis from adult (12 months) and aged (3.5 years) tree shrews. Left, male tree shrews; right, female tree shrews. Scale bar, 1 mm. **c** Three-point bending measurement of tibial maximum load, *n* = 6 per group. **d** Quantitative micro-CT analysis (**b**) of trabecular bone mass in femurs, *n* = 6 − 8 per group. **e** Representative H&E and Saf-O/FG staining of trabecular bone in femurs from adult and aged tree shrews. Scale bar, 500 μm. Data are expressed as the mean ± SD, *P < 0.05; **P < 0.01; ***P < 0.001; ****P < 0.000 1; ns*, P* non-significant

We further analyzed skeletal aging in tree shrews. In female tree shrews, compared with the adult group (12 months), the aged group (3.5 years) displayed significant reductions of BMD, BV/TV, connectivity density (Conn.D), and bone trabecular number (Tb.N), exhibiting osteoporotic changes of both bone mass and bone microstructure (Fig. 2a, b, d). The maximum load in the aged group was significantly lower compared to the adult group (Fig. 2c). Furthermore, histologic staining of femur sections indicated that the trabecular bone was irregular and notably reduced in the aged female group (Fig. 2e). In contrast, in male tree shrews, no statistically-significant differences in microstructural properties (BMD, BV/TV, Tb.N) were detected between the adult and aged groups (Fig. 2d), with no visible structural alterations of bone trabeculae in the aged group (Fig. 2e).

All these results indicated that aged female tree shrews were more susceptible than males to osteoporotic changes, which is similar to observations in humans.^38^

### Tree shrews had more limited self-healing capacity than rats in the osteochondral defect models

**Fig. 3:**
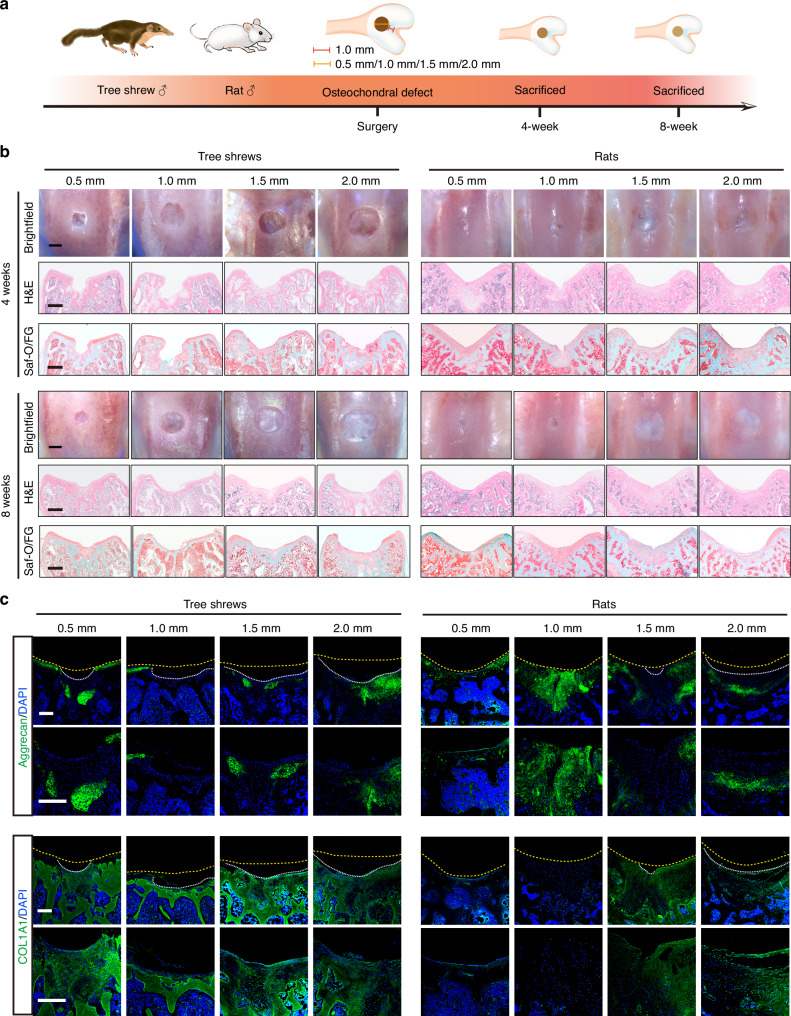
Tree shrews had more limited self-healing capacity than rats in the osteochondral defect models. **a** Schematic and timeline of establishment of an osteochondral defect model in tree shrews and rats. Cylindrical osteochondral defects of different sizes (0.5 mm, 1.0 mm, 1.5 mm, or 2.0 mm in diameter and 1.0 mm in depth) were created at 1.0 mm from the top of the intercondylar groove using dental drills. **b** Representative macroscopic appearance and staining of articular cartilage with H&E, and Saf-O/FG in tree shrews (left) and rats (right) at 4 weeks and 8 weeks post-surgery (top: 4 weeks, bottom: 8 weeks). Scale bar, 500 μm. **c** Representative IF images of aggrecan and COL1A1 in tree shrews (left) and rats (right) at 8 weeks post-surgery (top: aggrecan, bottom: COL1A1). Scale bar, 200 μm

Due to its limited healing capacity, cartilage injuries, particularly those affecting the articular cartilage, are predisposed to inducing or exacerbating OA.^39^ In this section, osteochondral defects of different sizes were created in tree shrews and their spontaneous healing capacities were investigated. We also included rats, which have similar joint size to tree shrews, as a reference for comparison (Fig. 3a). Four weeks after surgery, clear defects were still observed in all the groups of tree shrews with a little aggrecan detected at the edges of the defect areas (Figs. 3b, S2a), while in rats, different amounts of newly-formed tissue ingrowth could be observed macroscopically with some positive staining of aggrecan in the osteochondral defect area (Figs. 3b, S2b). Eight weeks after surgery, the osteochondral defect area in rats was filled with newly-formed tissue, the surface was flat, and well fused with the surrounding normal cartilage tissue. More detailed analyses revealed that 0.5 mm and 1.0 mm defects in rats were filled with translucent repair tissue that exhibited positive Safranin O and aggrecan staining, as well as negative COL1A1 staining. Meanwhile, 1.5 mm and 2.0 mm defects in rats were mainly filled with white-colored tissue with negative staining for Safranin O, and undetectable or minimal aggrecan staining, while COL1A1 was strongly expressed in the newly-formed tissue (Fig. 3b, c). In contrast, in tree shrews, defect areas of all sizes were filled with negligible fibrous tissues with a concave surface which remained below the height of normal tissue by the end of week eight (Fig. 3b). Most tissue in the defect area was stained positive for COL1A1, with little or no staining for aggrecan, suggesting the formation of heterogeneous fibrocartilage (Fig. 3c).

Taken together, these findings suggest that, compared with rapid spontaneous healing of the joint surface after injury, as observed in rats, articular cartilage in tree shrews resembled human cartilage with limited self-healing capacity.

### An OA model with progressive destruction of the articular cartilage was established in tree shrews

**Fig. 4:**
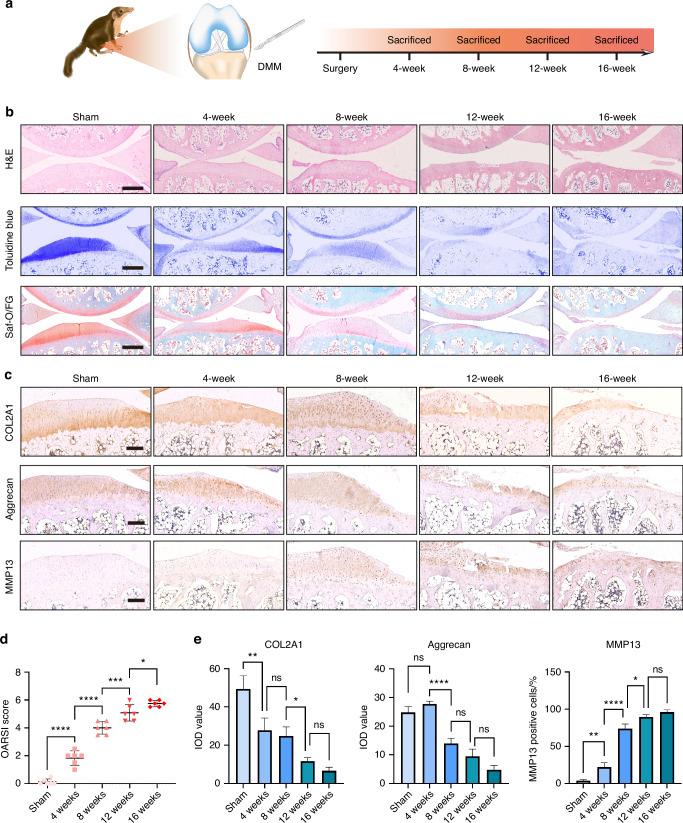
Establishment of an OA model with progressive destruction of the articular cartilage in tree shrews. **a** Schematic and timeline of establishment of an OA model in tree shrews by destabilization of the medial meniscus (DMM). **b** Representative staining of joint samples with H&E, Toluidine blue, and Saf-O/FG in sham and DMM-operated tree shrews at 4, 8, 12, and 16 weeks post-surgery. Scale bar, 500 μm. **c** Representative immunohistochemical (IHC) staining of joint samples with COL2A1, aggrecan, and MMP13 in sham and DMM-operated tree shrews at 4, 8, 12, and 16 weeks post-surgery. Scale bar, 200 μm. **d** OARSI score analysis in sham and DMM-operated tree shrews at 4, 8, 12, and 16 weeks post-surgery, *n* = 6 per group. **e** Quantification of IHC stains (c) for COL2A1, aggrecan, and MMP13 (*n* = 3). Data are expressed as the mean ± SD, *P < 0.05; **P < 0.01; ***P < 0.001; ****P < 0.000 1; ns, *P* non-significant

Destabilization of the medial meniscus (DMM) by surgically transecting the tibial ligament results in gradual deterioration of articular cartilage and subchondral bone changes, which is thought to recapitulate human pathological processes leading to OA.^40^ In this section, we performed DMM surgery in tree shrews as a model of post-traumatic OA (Fig. 4a). Four weeks after DMM, a rough surface with slight fibrillation was observed in the articular cartilage of the medial tibial plateau. Small cartilage lesions and markedly reduced proteoglycan staining across articular cartilage occurred at week eight. At 12 weeks and 16 weeks after DMM surgery, severe cartilage damage was evident, characterized by the loss of COL2A1 and aggrecan expression and a significant increase in MMP13 expression, indicating progression to moderate-to-severe stage OA (Fig. 4b, c, e). To further evaluate OA severity, the Safranin O and Fast Green sections were graded using the Osteoarthritis Research Society International (OARSI) scoring system with scores that range from 0 to a maximal score of 6.^41^ OARSI scores increased significantly over time, further indicating the progressive nature of OA in tree shrews (Fig. 4d).

These results revealed that a DMM-induced tree shrew model largely reproduced the pathological features of OA and provided theoretical support for using tree shrews as a potential model of human OA.

### Establishment of surgically-induced disc degeneration models in tree shrews

**Fig. 5:**
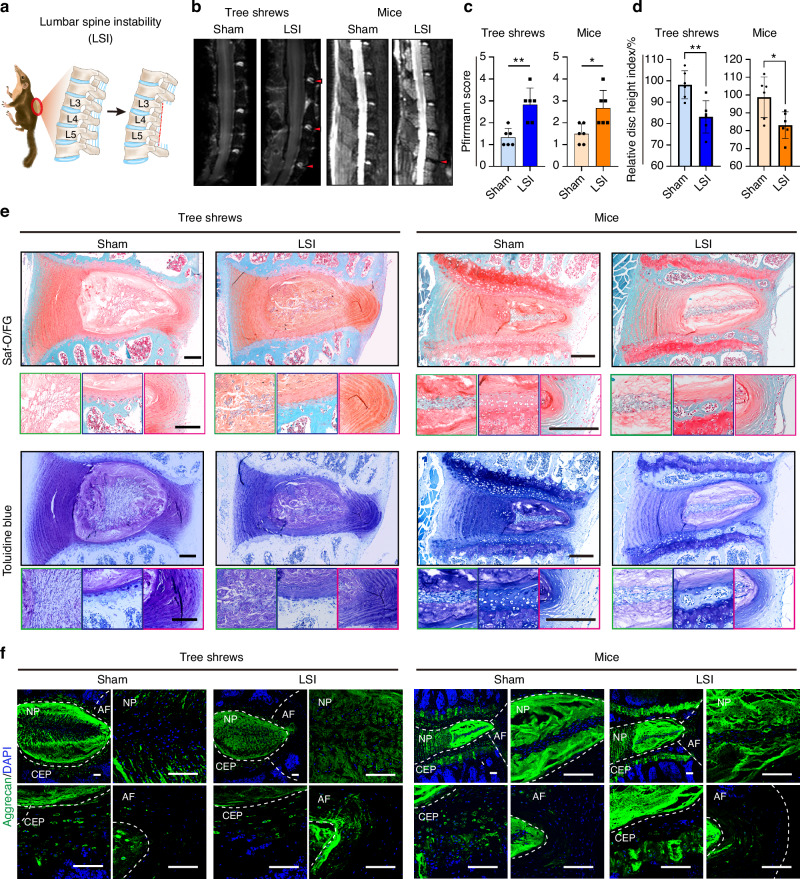
Establishment of a disc degeneration model by inducing lumbar spine instability in tree shrews. **a** Schematic of the lumbar spine instability model (LSI) in tree shrews. **b** Representative MRI images of the lumbar vertebrae of sham and LSI-operated animals at 12 weeks post-surgery. Left, tree shrews; right, mice. **c** Quantitative analysis of MRI data, *n* = 6 tree shrews and *n* = 6 mice. **d** Disc height index for LSI treatment. Left, tree shrews; right, mice, *n* = 6 tree shrews and *n* = 6 mice. **e** Representative staining of disc tissue with Saf-O/FG and Toluidine blue in sham and LSI-operated groups at 12 weeks post-surgery. Left, tree shrews; right, mice. Scale bar, 200 μm. **f** Representative IF images of aggrecan in sham and LSI-operated groups at 12 weeks post-surgery. Left, tree shrews; right, mice. Scale bar, 100 μm. Data are expressed as the mean ± SD, *P < 0.05; **P < 0.01; ***P < 0.001; ****P < 0.000 1; ns, *P* non-significant

The development of experimental models of IDD is essential considering that spontaneously-occurring IDD is limited and has the drawback of an inconsistent onset. Instability models induce slow and progressive disc degeneration with the advantages of keeping the disc intact and being close to the clinical condition.^42^ To create our model, we established lumbar spine instability (LSI) models in tree shrews (Fig. 5a) and mice. Twelve weeks later, MRI verified that both tree shrews and mice developed IDD after LSI treatment, exhibiting a decrease in disc space height, and higher Pfirrmann score (Fig. 5b–d). In mice, slight degenerative changes were observed, such as reduced numbers of NP cells and fragmentation of smaller cell clusters dispersed between the NP matrix rather than a larger single cluster. Tree shrews after LSI, on the other hand, developed more severe degenerative phenotypes, showing a loss of NP/AF demarcation, presence of round chondrocyte-like cells, discrete clusters with fewer NP cells, disrupted cartilage endplate, and reduced aggrecan staining in the NP and CEP (Fig. 5e, f).

**Fig. 6:**
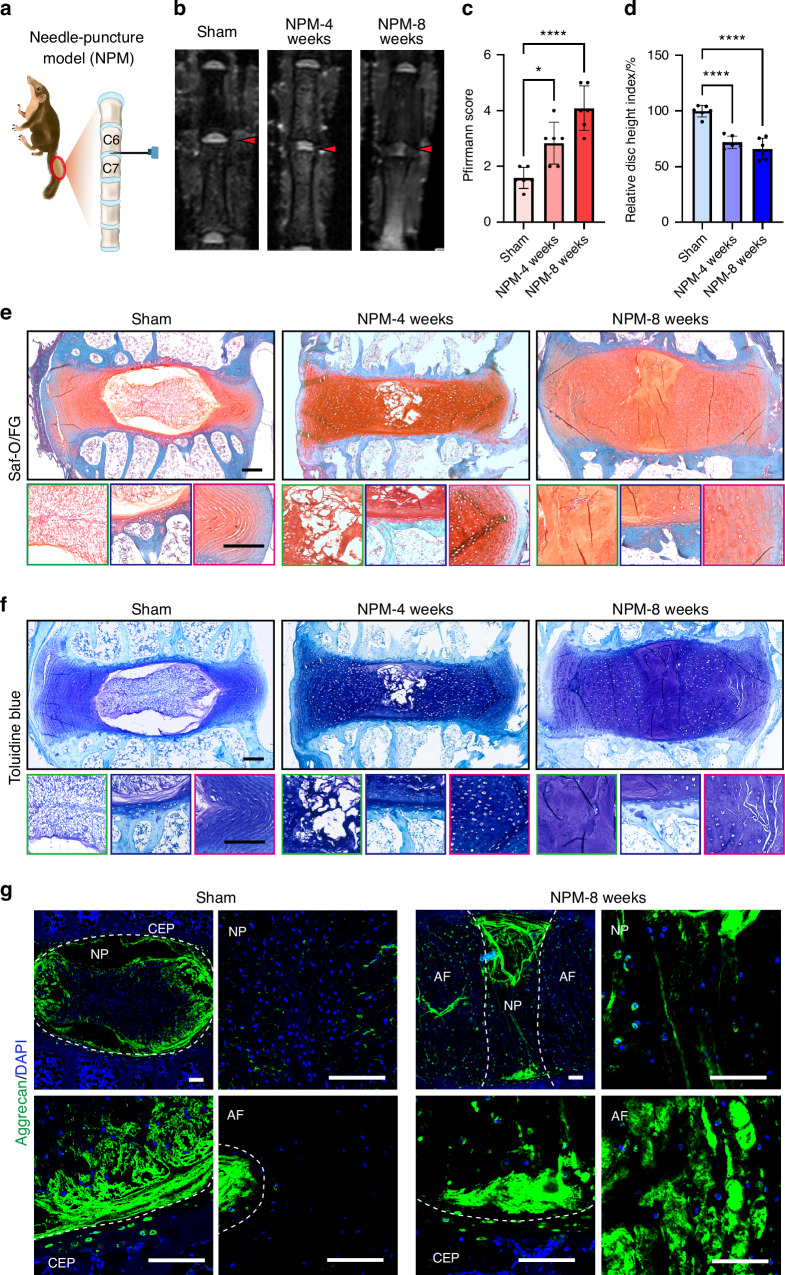
Establishment of a disc degeneration model by needle puncture in tree shrews. **a** Schematic of a needle-puncture model (NPM) in tree shrews. **b** Representative MRI images of the caudal vertebrae of sham and NPM-operated tree shrews at 4 and 8 weeks post-surgery. **c** Quantitative analysis of MRI data (*n* = 6). **d** Disc height index for needle-puncture treatment (*n* = 6). **e** Representative staining of disc tissue with Saf-O/FG in sham and NPM-operated tree shrews at 4 and 8 weeks post-surgery. Scale bar, 250 μm. **f** Representative staining of disc tissue with Toluidine blue in sham and NPM-operated tree shrews at 4 and 8 weeks post-surgery. Scale bar, 250 μm. **g** Representative IF images of aggrecan staining in sham and NPM-operated tree shrews at 4 and 8 weeks post-surgery. Scale bar, 100 μm

A needle puncture-induced disc degeneration model was also established in tree shrews and confirmed by MRI and histological analysis as early as four weeks after surgery (Fig. 6a–d). There were significant differences in tissue architecture and cell morphology in the NP, AF and CEP compartments by the end of eight weeks after puncture. Compared to the sham group, the degenerative caudal disc exhibited decrease in disc space height, disturbance of the boundary between the NP and AF, widening of the interlamellar space with chondrocyte-like tissue in parallel with a closely disappearing NP matrix (Fig. 6e, f). Moreover, aggrecan expression was decreased in CEP and increased in AF in the puncture group (Fig. 6g).

Taken together, these data revealed that LSI-induced IDD in tree shrews was apparent as slow and progressive disc degeneration, which approximates the clinical state; while a needle puncture tree shrew model led to the rapid development of IDD, providing a convenient platform for the study of IDD and evaluation of therapeutic interventions.

## Discussion

**Fig. 7:**
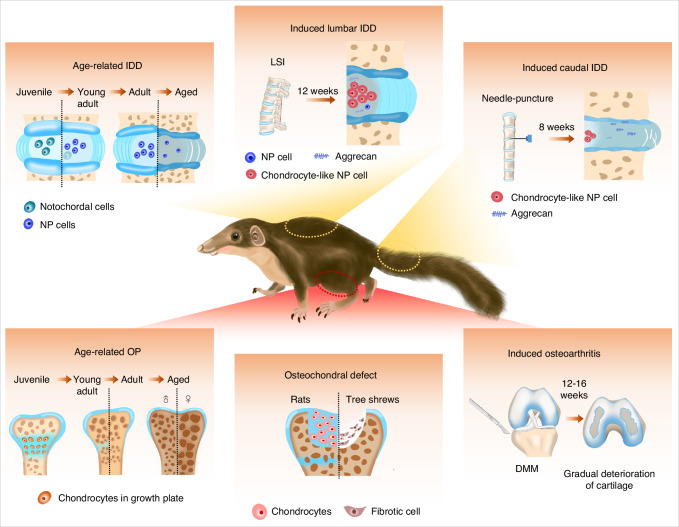
Schematic diagram of the tree shrew as a new animal model for studying common MSDs and disorders of aging including OP, IDD, OA, and osteochondral defects

The assessment of preclinical models of MSDs is paramount in the successful development and translation of innovative therapeutic approaches. In the present study, we investigated the potential of tree shrews as a new animal model for MSDs and aging. Our results revealed that the overall skeletal growth and bone metabolism of tree shrews are much closer to humans than those of rats/mice. Specifically, long bone epiphyseal and lumbar vertebral growth plates were closed in adult tree shrews. Aged tree shrews tended to suffer IDD and osteoporotic changes. We further constructed tree shrew models of osteochondral defects, OA, and IDD. The fact that tree shrews had poorer intrinsic healing potential of osteochondral defects, compared to the rat model, was a good indication that the tree shrew model was likely to better mimic the cartilage-related disease pathology seen in humans. Moreover, the tree shrew models of OA and IDD largely recapitulated the pathological features of human OA and disc degeneration, proved by histological and protein expression analyses, further broadening the application of the tree shrew as a viable animal model of MSDs (Fig. 7).

The major drawback of the rodent skeleton is that the growth plates do not undergo complete ossification, and they continue to grow lifelong after sexual maturity.^43,44^ Although there is no evidence to suggest that the slow continued growth of the murine skeleton affects the characteristics of age-related bone loss,^44,45^ concerns about its potential adverse effects remain. Here, our results revealed that longitudinal bone growth ceased at sexual maturity in tree shrews. Moreover, similar to what is observed in humans,^46^ with aging, female tree shrews had a greater decline in trabecular number than males. Thus, studies using tree shrews should be able to identify at least some of the factors that contribute to age-related bone loss in humans.

It has been reported that the presence of open growth plates in rodent models through advancing age likely increases intrinsic healing potential, giving confusing and potentially misleading results in tissue regeneration studies.^47^ We used an osteochondral defect model to investigate the spontaneous healing capacity of tree shrews and rats. Similar to a previous report,^48^ we found that 0.5 mm-diameter and 1.0 mm-diameter defects spontaneously healed, becoming filled with hyaline cartilage tissue in rats. In the tree shrews, only the 0.5 mm-diameter group exhibited fibrocartilage formation, while noticeable tissue defects persisted in the 1.0 to 2.0 mm diameter groups throughout the entire observation period. This finding suggested that tree shrews showed less intrinsic healing potential than rats, which may more closely approximate the human clinical situation, thus avoiding confused regeneration outcomes attributable to intrinsic healing potential. Although these results supported the innovative applied perspectives of tree shrews as a preclinical animal model in cartilage tissue engineering, tree shrew cartilage is still relatively thin compared to humans. Consequently, based on our results, we suggest that repair studies in tree shrews should use 1.0–2.0 mm diameter osteochondral defects, in which implantation is easier to perform and intrinsic repair processes predictably fail.

As the most common and chronic degenerative MSD, OA develops progressively over a span of decades with joint failure as its final outcome.^49^ The purpose of animal models is to controllably reproduce the pattern and progression of degenerative joint damage within a relatively short period, as too rapid progression to end-stage degeneration will miss important information on intermediate stages. In tree shrews, all the animals which underwent DMM surgery displayed chondrocyte matrix degradation, which gradually worsened over time. Moderate-to-severe OA was observed by the end of 12 weeks post-surgery. In mice, however, DMM has been shown to induce mild-to-moderate OA at 4 weeks, to moderate-to-severe OA at 8 weeks post-surgery.^40,50^ Despite the strength of a short experimental cycle in mice, it seems that tree shrews might more closely resemble the more slowly-progressive human OA and should allow for evaluation of disease modifying OA drugs. Our study provided the first theoretical support for the use of the tree shrew as an experimental OA model and revealed that a DMM-induced model of OA in tree shrews is highly efficient and reproducible, capable of mimicking physiological and pathological features of human OA to some degree. In the future, the tree shrew-OA model may form the basis of new strategies to optimize OA research.

Due to the complicated etiology and pathogenesis of disc degeneration, developing a suitable animal model is challenging. Several important characteristics must be taken into account when considering the potential suitability of an animal model, including variations in geometry, retained or altered notochordal cells, and persistent growth plates.^51^ Unlike large quadrupeds such as sheep, calves, and pigs in which the antero-posterior diameter of the vertebral endplates stays almost the same over the whole spine,^37^ tree shrews resemble humans in that this diameter steadily increases from the cervical to the lumbar region. Another similarity with human intervertebral discs that underpins the relevance of the tree shrew as a model is the loss of notochordal cells in the NP with age. As the presence or absence of notochordal cells is closely associated with IDD, results obtained from animals that retain notochordal cells well into adulthood such as rats and pigs may have little relevance to the adult human situation.^35,52^ From this point of view, the tree shrew model provides a more accurate representation of the IDD phenotype and interpretation/translation of results.

Age-related spontaneous IDD animal models have been reported in a limited number of species including nonhuman primates,^53^ sheep,^54^ and mice.^55^ In our study, we first described mild signs of IDD in 3.5 year-old tree shrews, characterized by a replacement of notochordal cells by mature nucleopulpocytes associated with an early decrease in aggrecan content. In particular, the loss of notochordal cells in the NP with age is similar to that observed in humans, further reinforcing the relevance of the tree shrew as a model. Age-related IDD was also detected in mice aged 18 months, with more severe degenerative characteristics. Notwithstanding these findings, considering that the open lumbar growth plates in rodents even with advancing age may influence the healing capacity of joint cartilaginous tissues, as well as the genetic difference between mice and humans, it remains to be determined whether these also relate to a differential susceptibility to the development of IDD using a mouse model.

The IDD induction technique is also an important consideration. Numerous experimental models have been developed with the aim of more accurately controlling the location and time course of degeneration as well as the amplitude of the induced injury.^42^ In our study, we established and characterized two models of IDD using LSI and needle puncture. The needle puncture tree shrew model led to rapid and severe development of IDD within four weeks. In comparison, LSI is more invasive and time-consuming, but the resultant slow and progressive disc degeneration shows greater similarity to the clinical state. Overall, we provide here a future reference for IDD studies in tree shrews based on different approaches depending on the experimental aims.

In conclusion, we have characterized age-related IDD and osteoporotic changes in aged tree shrews. Reproducible tree shrew models of osteochondral defects, surgery-induced OA and IDD were also developed, that present consistently with characteristics mimicking the spectrum of human pathology to some degree. Given that tree shrews have a much closer relationship to primates than rodents, we believe this animal model holds substantial potential for application in preclinical drug testing, disease modeling, and biomaterial research. Our work paved the way for the development of the tree shrew as a new animal model for the study of MSDs and aging.

## Materials and methods

### Animals and design

This study was approved by the Ethical Committee for Animal Research of Southern Medical University, and conducted according to guidelines from the Ministry of Science and Technology of China. Wild-type tree shrews were obtained from the Animal Center, Kunming Medical University, China. To study skeletal growth and aging, tree shrews and C57BL/6 mice of various ages were investigated, and the specific ages are outlined in each study. OA and IDD models were created using non-castrated adult male tree shrews (10–12 months, 150–180 g) or male C57BL/6 mice (3–4 months, 25–30 g). The osteochondral defect model was created using non-castrated adult male tree shrews (10–12 months, 150–180 g) and non-castrated male Sprague-Dawley (SD) rats (3 months, 180–200 g). The tree shrews (single cage housing) and rats (two rats per cage) were raised and maintained under standard conditions under a 12 h light/12 h dark cycle.

### Assessment of skeletal growth and aging

Tree shrews at different ages were used for assessment of their skeletal growth and aging. Males and females tree shrews at 20 and 45 days of age, 4.5 months of age, 12 months of age, and 3.5 years (*n* = 6 − 8) were euthanized and dissected. The femurs, tibias, and lumbar vertebrae (lumbar 4−sacrum 1) were immediately harvested and cleaned of soft tissue. The tibial bones were harvested for bone biomechanical examination. The femoral bones were extracted for micro-CT scanning and histology analysis. The vertebrae were excised for histology and MRI analysis. The lumbar vertebrae from male C57BL/6 mice at different ages (21 days; 3 months; 6 months; 18 months. *n* = 6) were also analyzed for comparison.

### Osteoarthritis model

The OA model in tree shrews was surgically induced by destabilization of the medial meniscus (DMM) following a previously-reported protocol.^40^ Tree shrews were deeply anesthetized with 4% isoflurane, and then maintained at 2% isoflurane at a rate of 2 L/min using an anesthesia machine (RWD Life Science Co., Ltd., Shenzhen, China). A 1-cm longitudinal incision was made over the right distal patella to the proximal tibial plateau. After opening the knee joint capsule, the fat pad was bluntly dissected over the intercondylar area to expose the meniscotibial ligament of the medial meniscus. Then, the medial meniscotibial ligament was transected with a scalpel blade to destabilize the joint. The joint capsule was closed with a 6-0 absorbable suture. The ligament was only exposed but not transected in the sham group. The tree shrews were sacrificed at 4, 8, 12, and 16 weeks after surgery.

### Osteochondral defect model

Tree shrews and rats were deeply anesthetized with 4% isoflurane, and then maintained at 2% isoflurane at a rate of 2 L/min using the anesthesia machine as described above. A small incision in the articular capsule was made along the patella and patellar tendon, and the patella was dislocated to the lateral side. An osteochondral defect of different sizes (diameter: 0.5 mm, 1.0 mm, 1.5 mm or 2.0 mm, depth: 1.0 mm) was created 1.0 mm from the top of the intercondylar groove using a dental drill, then the patella was repositioned and the joint capsule was closed with a 6-0 absorbable suture. In the sham group, an incision was created in the same way to expose the patella but no defects were created. The animals were sacrificed at 4 or 8 weeks after surgery.

### Intervertebral disc degeneration model

The lumbar spine instability model in tree shrews and mice was established following previously-described methods.^56,57^ After anesthesia, tree shrews and mice were placed in a prone position, and a longitudinal incision was created in the lower back region to expose the lumbar regions of the vertebral column. The lumbar 3rd–5th (L3–L5) spinous processes along with the supraspinous and interspinous ligaments were resected to induce instability of the lumbar spine. In the sham group, the posterior paravertebral muscles from vertebrae L3–L5 were detached but without resecting the spinous processes or ligaments. The animals were sacrificed at 12 weeks after surgery.

The needle puncture-induced IDD model was established in tree shrews following previous reports.^58,59^ After shaving and sterilizing the tail skin, a 29-gauge needle was stabbed into the tail disc at the Co6/Co7 level. The needle was vertically inserted into the disc to 5 mm depth to pass through the inner AF, and then the needle was rotated 360° and kept in the disc for 60 s before removal. In the sham group, tree shrews were placed under anesthesia but not subjected to needle stab injury. The animals were sacrificed at 4 and 8 weeks after the surgery.

### MRI analysis

MRI study was performed using a PharmaScan 7.0 T system (BioSpin MRI GmbH Bruker, Ettlingen, Germany). Sagittal T2-weighted images of the lumbar or caudal spine were acquired and were assessed by blinded orthopedic researchers using the Pfirrmann MRI grading system.^60^

### Micro-CT analysis

The femurs from tree shrews were subjected to high-resolution micro-CT analysis using a micro-CT imaging system (Siemens, Munich, Germany). The three-dimensional (3D) images were reconstructed and analyzed using Siemens Multimodal 3D Visualization software (Siemens) for quantitative parameters of trabecular and cortical bone, including the relative bone density, bone volume (BV/TV), bone surface (BS/TV), trabecular number (Tb.N), trabecular thickness (Tb.Th), trabecular separation (Tb.Sp) and cortical thickness (Ct.Th).

### Biomechanical examination

The mechanical properties of the tibias from tree shrews of the different groups were evaluated using a three-point bending mechanical strength device (AG-IS, Shimadzu, Japan). The fresh bones were wrapped in normal saline-soaked gauze and then centered longitudinally with the anterior surface supported on the two lower holding devices (2 cm distance). A third rounded bar was placed at the medial of the diaphysis with a 10 N preload strength, and then a constant displacement rate of 1 mm/min was applied until the tibia fractured. The mechanical data were collected and analyzed to determine the maximum load.

### Histology, immunohistochemistry (IHC) and immunofluorescence (IF)

Femurs and vertebrae were fixed in 4% paraformaldehyde, demineralized in 0.5 mol/L EDTA for 2 months until bones were pliable, then embedded in paraffin for sectioning at a thickness of 4 μm. Sections were incubated on slides (37 °C, overnight), deparaffinized, rehydrated, and used for histology, IHC, and/or IF analysis.

For hematoxylin and eosin (H&E) staining, the tissue sections were stained with hematoxylin (Sigma-Aldrich, St. Louis, MO, USA) for 5 min, followed by 30 s of staining with eosin (Wako Chemicals, Richmond, VA, USA). For Safranin-O/Fast Green staining, sections were subjected to Fast Green (Sigma-Aldrich) staining for 2 min and subsequently stained with Safranin-O (Sigma-Aldrich) for 8 min at room temperature. Toluidine blue staining was performed according to the manufacturer’s instructions (Sigma-Aldrich). After staining, the sections were dehydrated, cleared, and mounted. Images were acquired using a slide scanner (VS200, Olympus, Tokyo, Japan) and further processed using OlyVIA VS200.

For IF staining, sections were subjected to rehydration and antigen retrieval by incubation in 10 mmol/L citric acid buffer (pH 6) overnight at 60 °C. Then samples were permeabilized with 0.3% Triton X-100 for 15 min and blocked with 1% BSA for 30 min. Sections were then incubated with primary antibodies against aggrecan (1:200, Proteintech, Rosemont, IL, USA; 13880-1-AP) or COL1A1 (1:200, Cell Signaling Technology, Danvers, MA, USA; 91144S), overnight at 4 °C. Next day, sections were washed and incubated with the secondary antibody Alexa Fluor 488 (1:1 000, Abcam, ab150073) for 1 h at room temperature in the dark, then counterstained with 4′,6-diamidino-2-phenylindole (DAPI) (Sigma-Aldrich, D8417).

For IHC staining, sections were incubated in 10 mmol/L citric acid buffer (pH 6) overnight at 60 °C for rehydration and antigen retrieval, quenched with 3% hydrogen peroxide, and then permeabilized with 0.5% Triton X-100. Next, after washing with PBS, the sections were blocked in 5% normal goat serum at room temperature for 1 h. Sections were then incubated with primary antibodies against COL2A1 (1:200, Abcam, Cambridge, MA, USA, ab34712), aggrecan (1:200, Proteintech, Rosemont, IL, USA; 13880-1-AP) and MMP13 (1:200, Proteintech, 18165-1-AP) overnight at 4 °C. Next day the slides were incubated with secondary biotinylated goat anti-rabbit antibody (1:300, Jackson ImmunoResearch Laboratories, West Grove, PA, USA) for one hour at room temperature followed by chromogen DAB staining (ZSGB-BIO Co., Ltd., Beijing, China, cat ZLI-9018) and hematoxylin counterstaining. After staining, the sections were dehydrated, cleared, and mounted. Images were acquired using a slide scanner (VS200, Olympus) and further processed using OlyVIA VS200.

### Statistical analysis

All data are expressed as the mean ± standard deviation (SD) with sample sizes indicated in the legends. All data met the assumptions of the test with regard to the normality and homogeneity of variance. Statistical comparisons between two groups were assessed using Student’s t-tests. When comparing multiple groups, one-way analysis of variance was used. Statistically-significant differences were considered at *P*-values < 0.05 (*P < 0.05; **P < 0.01; ***P < 0.001; ****P < 0.000 1; ns, *P* non-significant). All statistical analyses were performed utilizing Prism 9 software (GraphPad, San Diego, CA, USA).

## Supplementary information

Supplementary Materials for Tree shrew as a new animal model for musculoskeletal disorders and aging

## References

[1] Wen, Z. et al. Insights into the underlying pathogenesis and therapeutic potential of endoplasmic reticulum stress in degenerative musculoskeletal diseases. *Mil. Med. Res.* **10**, 1 (2023). 37941072 10.1186/s40779-023-00485-5 PMC10634069

[2] Hartvigsen, J. et al. What low back pain is and why we need to pay attention. *Lancet* **391**, 2356 (2018). 29573870 10.1016/S0140-6736(18)30480-X

[3] Fine, N. et al. Intervertebral disc degeneration and osteoarthritis: a common molecular disease spectrum. *Nat. Rev. Rheumatol.* **19**, 136 (2023). 36702892 10.1038/s41584-022-00888-z

[4] Hunter, D. J. & Bierma-Zeinstra, S. Osteoarthritis. *Lancet* **393**, 1745 (2019). 31034380 10.1016/S0140-6736(19)30417-9

[5] Latourte, A., Kloppenburg, M. & Richette, P. Emerging pharmaceutical therapies for osteoarthritis. *Nat. Rev. Rheumatol.* **16**, 673 (2020). 33122845 10.1038/s41584-020-00518-6

[6] Song, C. et al. Bioenergetic dysfunction in the pathogenesis of intervertebral disc degeneration. *Pharm. Res.* **202**, 107119 (2024). 10.1016/j.phrs.2024.107119 38417775

[7] Kwon, H. et al. Surgical and tissue engineering strategies for articular cartilage and meniscus repair. *Nat. Rev. Rheumatol.* **15**, 550 (2019). 31296933 10.1038/s41584-019-0255-1 PMC7192556

[8] Ying, Y. et al. Recent advances in the repair of degenerative intervertebral disc for preclinical applications. *Front. Bioeng. Biotech.* **11**, 1259731 (2023). 10.3389/fbioe.2023.1259731 PMC10557490 37811372

[9] Colella, F. et al. Drug delivery in intervertebral disc degeneration and osteoarthritis: Selecting the optimal platform for the delivery of disease-modifying agents. *J. Control Release* **328**, 985 (2020). 32860929 10.1016/j.jconrel.2020.08.041

[10] Chevalier, X., Eymard, F. & Richette, P. Biologic agents in osteoarthritis: hopes and disappointments. *Nat. Rev. Rheumatol.* **9**, 400 (2013). 23545735 10.1038/nrrheum.2013.44

[11] Leenaars, C. et al. Animal to human translation: a systematic scoping review of reported concordance rates. *J. Transl. Med.* **17**, 223 (2019). 31307492 10.1186/s12967-019-1976-2 PMC6631915

[12] Gullbrand, S. E. et al. A large animal model that recapitulates the spectrum of human intervertebral disc degeneration. *Osteoarthr. Cartil.* **25**, 146 (2017). 10.1016/j.joca.2016.08.006 PMC5182186 27568573

[13] Zhang, Z. et al. Animal models for glucocorticoid-induced postmenopausal osteoporosis: An updated review. *Biomed. Pharmacother.* **84**, 438 (2016). 27685786 10.1016/j.biopha.2016.09.045

[14] Little, C. B. & Hunter, D. J. Post-traumatic osteoarthritis: from mouse models to clinical trials. *Nat. Rev. Rheumatol.* **9**, 485 (2013). 23689231 10.1038/nrrheum.2013.72

[15] Yilmaz, D., Mathavan, N., Wehrle, E., Kuhn, G. A. & Muller, R. Mouse models of accelerated aging in musculoskeletal research for assessing frailty, sarcopenia, and osteoporosis - a review. *Ageing Res. Rev.* **93**, 102118 (2024). 37935249 10.1016/j.arr.2023.102118

[16] Beagan, M. et al. The potential of sheep in preclinical models for bone infection research - A systematic review. *J. Orthop. Transl.* **45**, 120 (2024). 10.1016/j.jot.2024.02.002 PMC10960093 38524868

[17] Frisbie, D. D., Cross, M. W. & McIlwraith, C. W. A comparative study of articular cartilage thickness in the stifle of animal species used in human pre-clinical studies compared to articular cartilage thickness in the human knee. *Vet. Comp. Orthopaed.* **19**, 142 (2006). 16971996

[18] Cao, J., Yang, E. B., Su, J. J., Li, Y. & Chow, P. The tree shrews: adjuncts and alternatives to primates as models for biomedical research. *J. Med. Primatol.* **32**, 123 (2003). 12823622 10.1034/j.1600-0684.2003.00022.x

[19] Yao, Y. G. Creating animal models, why not use the Chinese tree shrew (Tupaia belangeri chinensis)? *Zool. Res.* **38**, 118 (2017). 28585435 10.24272/j.issn.2095-8137.2017.032 PMC5460080

[20] Fan, Y. et al. Genome of the Chinese tree shrew. *Nat. Commun.* **4**, 1426 (2013). 23385571 10.1038/ncomms2416

[21] Fan, Y., Yu, D. & Yao, Y. G. Tree shrew database (TreeshrewDB): a genomic knowledge base for the Chinese tree shrew. *Sci. Rep.-Uk* **4**, 7145 (2014). 10.1038/srep07145 PMC5382678 25413576

[22] Fan, Y. et al. Chromosomal level assembly and population sequencing of the Chinese tree shrew genome. *Zool. Res.* **40**, 506 (2019). 31418539 10.24272/j.issn.2095-8137.2019.063 PMC6822927

[23] Ye, M. S. et al. Comprehensive annotation of the Chinese tree shrew genome by large-scale RNA sequencing and long-read isoform sequencing. *Zool. Res.* **42**, 692 (2021). 34581030 10.24272/j.issn.2095-8137.2021.272 PMC8645884

[24] Li, C. H. et al. Long-term propagation of tree shrew spermatogonial stem cells in culture and successful generation of transgenic offspring. *Cell Res.* **27**, 241 (2017). 28008926 10.1038/cr.2016.156 PMC5339851

[25] Khanal, S., Norton, T. T. & Gawne, T. J. Limited bandwidth short-wavelength light produces slowly-developing myopia in tree shrews similar to human juvenile-onset myopia. *Vis. Res.* **204**, 108161 (2023). 36529048 10.1016/j.visres.2022.108161 PMC9974583

[26] She, Z., Ward, A. H. & Gawne, T. J. The effects of ambient narrowband long-wavelength light on lens-induced myopia and form-deprivation myopia in tree shrews. *Exp. Eye Res.* **234**, 109593 (2023). 37482282 10.1016/j.exer.2023.109593 PMC10529043

[27] Ku, H. et al. Myopia development in tree shrew is associated with chronic inflammatory reactions. *Curr. Issues Mol. Biol.* **44**, 4303 (2022). 36135208 10.3390/cimb44090296 PMC9498061

[28] Yi, J. et al. Co-delivery of Cas9 mRNA and guide RNAs edits hepatitis B virus episomal and integration DNA in mouse and tree shrew models. *Antivir. Res.* **215**, 105618 (2023). 37142191 10.1016/j.antiviral.2023.105618

[29] Li, Y. T., Wu, H. L. & Liu, C. J. Molecular Mechanisms and animal models of HBV-related hepatocellular carcinoma: with emphasis on metastatic tumor antigen 1. *Int. J. Mol. Sci.* **22**, 9380 (2021). 34502289 10.3390/ijms22179380 PMC8431721

[30] Schmelting, B. et al. Agomelatine in the tree shrew model of depression: effects on stress-induced nocturnal hyperthermia and hormonal status. *Eur. Neuropsychopharm* **24**, 437 (2014). 10.1016/j.euroneuro.2013.07.010 23978391

[31] Wei, S. et al. Dynamic changes in DNA demethylation in the tree shrew (Tupaia belangeri chinensis) brain during postnatal development and aging. *Zool. Res.* **38**, 96 (2017). 28409505 10.24272/j.issn.2095-8137.2017.013 PMC5396032

[32] Vollmayr, B., Mahlstedt, M. M. & Henn, F. A. Neurogenesis and depression: what animal models tell us about the link. *Eur. Arch. Psy Clin. N.* **257**, 300 (2007). 10.1007/s00406-007-0734-2 17401725

[33] Wang, Y. et al. Establishment of an osteoporosis model in tree shrews by bilateral ovariectomy and comprehensive evaluation. *Exp. Ther. Med.* **17**, 3644 (2019). 30988748 10.3892/etm.2019.7339 PMC6447825

[34] Chen, Q. et al. A tree shrew model for steroid-associated osteonecrosis. *Zool. Res.* **41**, 564 (2020). 32738109 10.24272/j.issn.2095-8137.2020.061 PMC7475020

[35] Colombier, P., Clouet, J., Hamel, O., Lescaudron, L. & Guicheux, J. The lumbar intervertebral disc: from embryonic development to degeneration. *Jt. Bone Spine* **81**, 125 (2014). 10.1016/j.jbspin.2013.07.012 23932724

[36] Kawashima, T., Thorington, R. J., Bohaska, P. W. & Sato, F. Variability and constraint of vertebral formulae and proportions in colugos, tree shrews, and rodents, with special reference to vertebral modification by aerodynamic adaptation. *Folia Morphol.* **77**, 44 (2018). 10.5603/FM.a2017.0064 28703847

[37] Alini, M. et al. Are animal models useful for studying human disc disorders/degeneration? *Eur. Spine J.* **17**, 2 (2008). 17632738 10.1007/s00586-007-0414-y PMC2365516

[38] Zhang, Y. Y. et al. Insights and implications of sexual dimorphism in osteoporosis. *Bone Res.* **12**, 8 (2024). 38368422 10.1038/s41413-023-00306-4 PMC10874461

[39] Armiento, A. R., Alini, M. & Stoddart, M. J. Articular fibrocartilage - why does hyaline cartilage fail to repair? *Adv. Drug Deliv. Rev.* **146**, 289 (2019). 30605736 10.1016/j.addr.2018.12.015

[40] Glasson, S. S., Blanchet, T. J. & Morris, E. A. The surgical destabilization of the medial meniscus (DMM) model of osteoarthritis in the 129/SvEv mouse. *Osteoarthr. Cartil.* **15**, 1061 (2007). 10.1016/j.joca.2007.03.006 17470400

[41] Glasson, S. S., Chambers, M. G., Van Den Berg, W. B. & Little, C. B. The OARSI histopathology initiative - recommendations for histological assessments of osteoarthritis in the mouse. *Osteoarthr. Cartil.* **18**, S17 (2010). 10.1016/j.joca.2010.05.025 20864019

[42] Poletto, D. L., Crowley, J. D., Tanglay, O., Walsh, W. R. & Pelletier, M. H. Preclinical in vivo animal models of intervertebral disc degeneration. Part 1: a systematic review. *Jor Spine* **6**, e1234 (2023). 36994459 10.1002/jsp2.1234 PMC10041387

[43] Brent, M. B. Pharmaceutical treatment of bone loss: from animal models and drug development to future treatment strategies. *Pharm. Therapeut* **244**, 108383 (2023). 10.1016/j.pharmthera.2023.108383 36933702

[44] Jilka, R. L. The relevance of mouse models for investigating age-related bone loss in humans. *J. Gerontol. A-Biol.* **68**, 1209 (2013). 10.1093/gerona/glt046 PMC3779631 23689830

[45] Koh, N., Miszkiewicz, J. J., Fac, M. L., Wee, N. & Sims, N. A. Preclinical rodent models for human bone disease, including a focus on cortical bone. *Endocr. Rev.* **45**, 493 (2024). 38315213 10.1210/endrev/bnae004 PMC11244217

[46] Khosla, S. et al. Effects of sex and age on bone microstructure at the ultradistal radius: a population‐based noninvasive in vivo assessment. *J. Bone Miner. Res.* **21**, 124 (2006). 16355281 10.1359/JBMR.050916 PMC1352156

[47] Chu, C. R., Szczodry, M. & Bruno, S. Animal models for cartilage regeneration and repair. *Tissue Eng. Part B-Re* **16**, 105 (2010). 10.1089/ten.teb.2009.0452 PMC3121784 19831641

[48] Katagiri, H., Mendes, L. F. & Luyten, F. P. Definition of a critical size osteochondral knee defect and its negative effect on the surrounding articular cartilage in the rat. *Osteoarthr. Cartil.* **25**, 1531 (2017). 10.1016/j.joca.2017.05.006 PMC5754326 28506841

[49] Martel-Pelletier, J. et al. Osteoarthritis. *Nat. Rev. Dis. Prim.* **2**, 16072 (2016). 27734845 10.1038/nrdp.2016.72

[50] Kim, S. et al. Tankyrase inhibition preserves osteoarthritic cartilage by coordinating cartilage matrix anabolism via effects on SOX9 PARylation. *Nat. Commun.* **10**, 4818 (2019). 31653858 10.1038/s41467-019-12910-2 PMC6814715

[51] Daly, C., Ghosh, P., Jenkin, G., Oehme, D. & Goldschlager, T. A review of animal models of intervertebral disc degeneration: pathophysiology, regeneration, and translation to the clinic. *Biomed. Res. Int.* **2016**, 5952165 (2016). 27314030 10.1155/2016/5952165 PMC4893450

[52] Li, Y. et al. Notochordal cells: a potential therapeutic option for intervertebral disc degeneration. *Cell Proliferat* **57**, e13541 (2024). 10.1111/cpr.13541 PMC10849793 37697480

[53] Nuckley, D. J. et al. Intervertebral disc degeneration in a naturally occurring primate model: radiographic and biomechanical evidence. *J. Orthop. Res.* **26**, 1283 (2008). 18404651 10.1002/jor.20526

[54] Bouhsina, N. et al. Correlation between magnetic resonance, X-ray imaging alterations and histological changes in an ovine model of age-related disc degeneration. *Eur. Cells Mater.* **42**, 166 (2021). 10.22203/eCM.v042a13 34558056

[55] Ohnishi, T., Sudo, H., Tsujimoto, T. & Iwasaki, N. Age‐related spontaneous lumbar intervertebral disc degeneration in a mouse model. *J. Orthop. Res.* **36**, 224 (2018). 28631843 10.1002/jor.23634

[56] Miyamoto, S., Yonenobu, K. & Ono, K. Experimental cervical spondylosis in the mouse. *Spine* **16**, S495 (1991). 1801260 10.1097/00007632-199110001-00008

[57] Bian, Q. et al. Mechanosignaling activation of TGFbeta maintains intervertebral disc homeostasis. *Bone Res.* **5**, 17008 (2017). 28392965 10.1038/boneres.2017.8 PMC5360159

[58] Chan, A. K. et al. Pulsed electromagnetic fields reduce acute inflammation in the injured rat-tail intervertebral disc. *Jor Spine* **2**, e1069 (2019). 31891118 10.1002/jsp2.1069 PMC6920683

[59] Zhang, W. et al. Cytosolic escape of mitochondrial DNA triggers cGAS-STING-NLRP3 axis-dependent nucleus pulposus cell pyroptosis. *Exp. Mol. Med.* **54**, 129 (2022). 35145201 10.1038/s12276-022-00729-9 PMC8894389

[60] Pfirrmann, C. W., Metzdorf, A., Zanetti, M., Hodler, J. & Boos, N. Magnetic resonance classification of lumbar intervertebral disc degeneration. *Spine* **26**, 1873 (2001). 11568697 10.1097/00007632-200109010-00011

