# Supplementary material for: Tree shrew as a new animal model for musculoskeletal disorders and aging
Source: Bone Res. 2025 Jan 2;13:5. doi: 10.1038/s41413-024-00367-z (PMC11697419; doi:10.1038/s41413-024-00367-z)
Supplement: Supplementary file 1 — Supplementary Materials for Tree shrew as a new animal model for musculoskeletal disorders and aging [file 41413_2024_367_MOESM1_ESM.docx]

**Supplementary Materials for**

**Tree shrew as a new animal model for musculoskeletal disorders and aging**

**The file includes:**

Figures. S1, S2

**
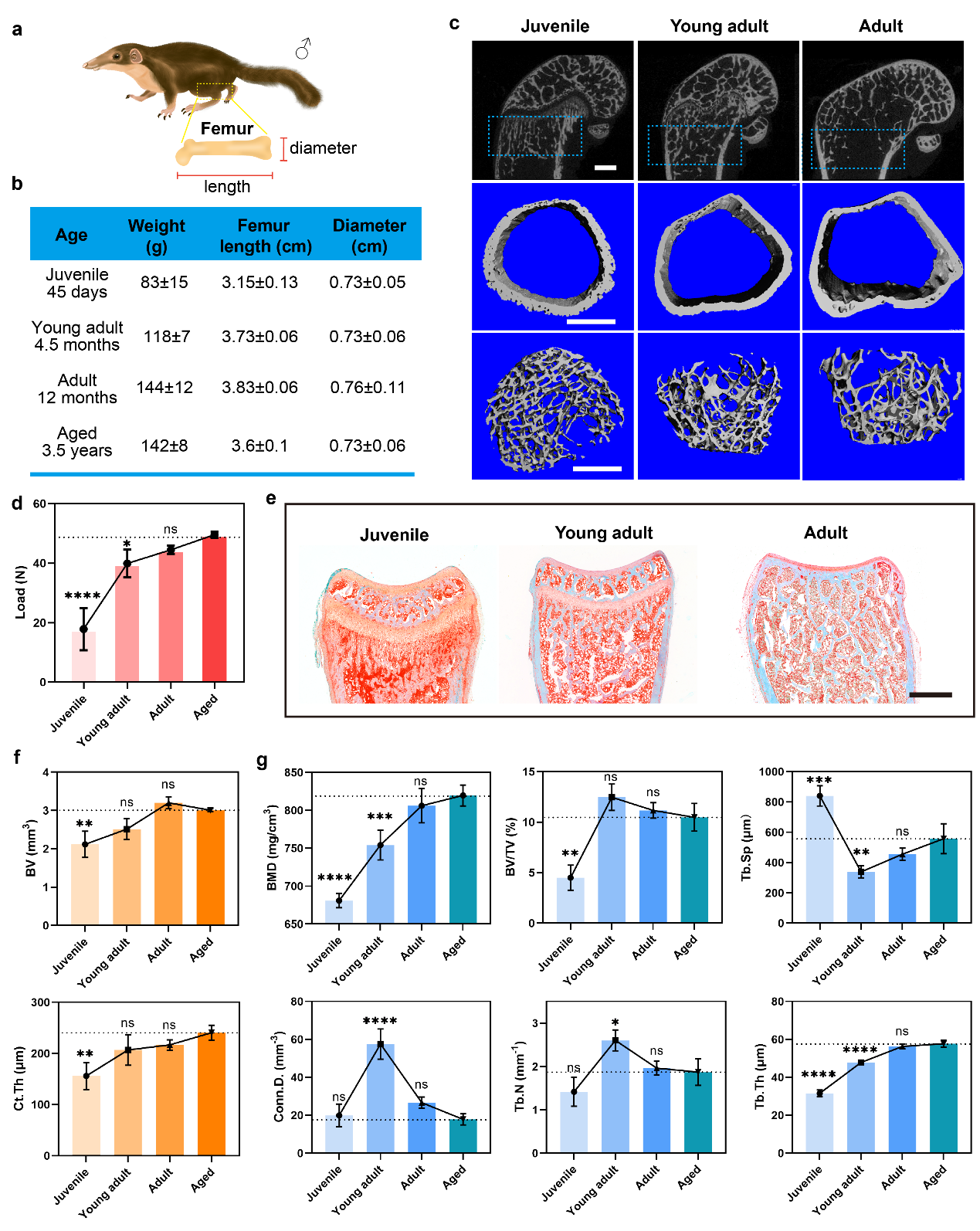
**

**Fig. S1** **Characterization of skeletal mineralization and growth-plate structure in tree shrews with aging, related to Fig. 2**

(a, b) The general information and morphological characterization of the femurs of male tree shrews at different ages (juvenile, 45 days; young adult, 4.5 months; adult, 12 months; aged, 3.5 years). (c) Representative micro-CT 3D reconstructed images showing the distal femoral metaphysis from male tree shrews at different ages. Scale bar, 1 mm. (d) Three-point bending measurement of tibial maximum load from male tree shrews at different ages, n = 3. (e) Representative Saf-O/FG staining of the femurs from male tree shrews at different ages. Scale bar, 1 mm. Quantitative micro-CT analysis of cortical bone (f) and trabecular bone (g) mass in femurs from male tree shrews at different ages, n=3. Data are expressed as the mean ± SD, **P < 0.05; **P < 0.01; ***P < 0.001; ****P < 0.0001*; *n.s., P* non-significant.


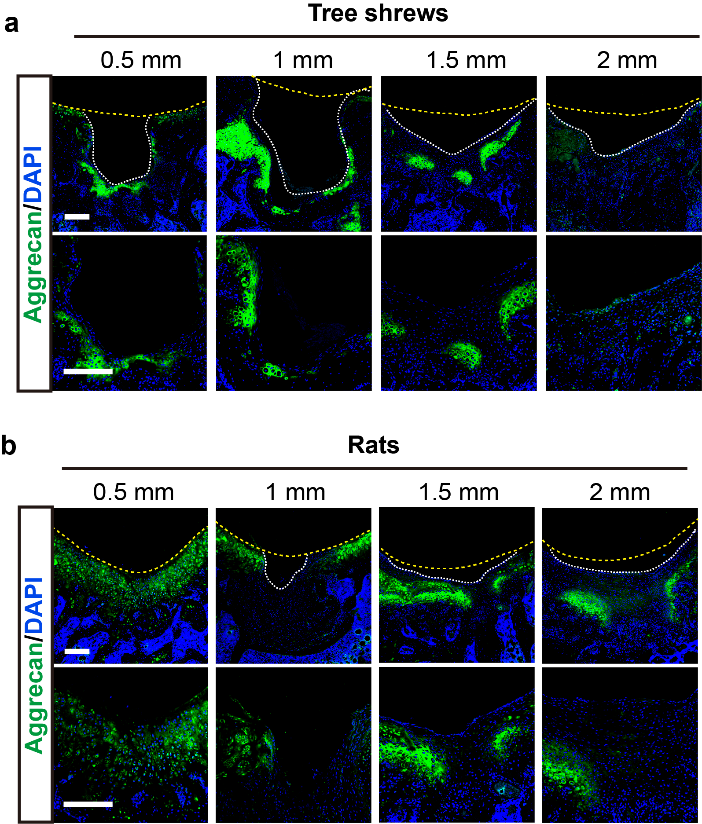


**Fig. S2** **The defect area in rats exhibited more aggrecan staining than those from tree shrews, related to Fig. 3**

(a) Representative IF images of aggrecan in tree shrews at 4 weeks post-surgery; n = 3 per group. Scale bar, 200 μm. (b) Representative IF images of aggrecan in rats at 4 weeks post-surgery. Scale bar, 200 μm.
